# Understanding the role of AMPA receptors in autism: insights from circuit and synapse dysfunction

**DOI:** 10.3389/fpsyt.2024.1304300

**Published:** 2024-01-30

**Authors:** Andres Jimenez-Gomez, Megan X. Nguyen, Jason S. Gill

**Affiliations:** ^1^ Neurodevelopmental Disabilities Program, Department of Neurology, Joe DiMaggio Children’s Hospital, Hollywood, FL, United States; ^2^ Department of Pediatrics, Division of Neurology & Developmental Neurosciences, Baylor College of Medicine, Houston, TX, United States; ^3^ Jan & Dan Duncan Neurologic Research Institute, Texas Children’s Hospital, Houston, TX, United States

**Keywords:** autism, AMPA, circuits, cerebro-cerebellar, ASD, SynGAP1, SHANK3, synaptopathy

## Abstract

Autism spectrum disorders represent a diverse etiological spectrum that converge on a syndrome characterized by discrepant deficits in developmental domains often highlighted by concerns in socialization, sensory integration, and autonomic functioning. Importantly, the incidence and prevalence of autism spectrum disorders have seen sharp increases since the syndrome was first described in the 1940s. The wide etiological spectrum and rising number of individuals being diagnosed with the condition lend urgency to capturing a more nuanced understanding of the pathogenic mechanisms underlying the autism spectrum disorders. The current review seeks to understand how the disruption of AMPA receptor (AMPAr)-mediated neurotransmission in the cerebro-cerebellar circuit, particularly in genetic autism related to SHANK3 or SYNGAP1 protein dysfunction function and autism associated with *in utero* exposure to the anti-seizure medications valproic acid and topiramate, may contribute to the disease presentation. Initially, a discussion contextualizing AMPAr signaling in the cerebro-cerebellar circuitry and microstructural circuit considerations is offered. Subsequently, a detailed review of the literature implicating mutations or deletions of *SHANK3* and *SYNGAP1* in disrupted AMPAr signaling reveals how bidirectional pathogenic modulation of this key circuit may contribute to autism. Finally, how pharmacological exposure may interact with this pathway, *via* increased risk of autism diagnosis with valproic acid and topiramate exposure and potential treatment of autism using AMPAr modulator perampanel, is discussed. Through the lens of the review, we will offer speculation on how neuromodulation may be used as a rational adjunct to therapy. Together, the present review seeks to synthesize the disparate considerations of circuit understanding, genetic etiology, and pharmacological modulation to understand the mechanistic interaction of this important and complex disorder.

## Introduction

Autism spectrum disorders (ASDs) comprise an etiologically heterogeneous group of disorders defined primarily by impaired social function and discrepant achievement of milestones in time and across developmental domains (1–3). Since being identified as a unique neurodevelopmental disorder in 1943, ASD has emerged as a prevalent and societally impactful neurodevelopmental disorder, currently affecting ~1.5% of the population (4, 5). Since epidemiological tracking began around 2000, the incidence of ASD has continued to rise, a trend that is likely driven by a variety of factors including increased awareness of the disorder by caretakers and clinicians, changes in diagnostic criteria, and altered state and federal regulations (6–8).

The clinical definition and presentation of ASD has undergone an expansion over the past decade, particularly following the re-categorization of autism and other pervasive developmental disorders under the broader “umbrella” of ASD in the American Diagnostic and Statistical Manual of Mental Health Disorders, 5th edition (DSM-5) (9). With this, the clinical evaluation of affected individuals has been defined through two primary categories of impact observed across a continuum of neurodevelopment, from a more “classic” perspective of severe/profound autism to the neurodivergent traits in those with “high functioning” autism:

1) Persistent difficulties with social communication and social interaction: clinically encompassing the oft described symptoms of decreased eye contact, limited verbal overtures, and nonverbal gesturing or understanding thereof (e.g., decreased response to name, pointing or lack of following another person pointing), and also inclusive of other more developmentally sophisticated communication challenges (e.g., ambiguous understanding of humor and linguistic nuances (10)).
2) Restricted and repetitive patterns of behaviors, activities, or interests: these stretch from the markedly atypical and perseverative stereotypies and sensory-seeking/aversive behaviors often documented in clinical literature and societal representations of ASD (e.g., hand-flapping, toe-walking, fascination with spinning objects), to traits that demand higher cognitive and communicative acquisition (e.g., extreme fascination with and research on esoteric subjects, or unusual and extensive collections of items (10)).

As noted, the clinical signs and symptoms, while covered under a single phenotypic ontology, have possible extreme variations between (and longitudinally within) individuals. This has harbored the discussion of both the possibility of a different categorization of “profound autism” (11) and recent studies on the “resolution” of the ASD diagnosis across the lifespan (12).

As noted, the diagnosis of ASD remains based on clinical evaluation rather than the presence of any given serum or imaging biomarker, which introduces a great degree of variability in application of diagnostic criteria and therefore in the diagnosis itself (6–8). Furthermore, the known etiological contributors to ASD diagnosis are broad, including single gene mutations with Mendelian inheritance (3); acquired and environmental influences in the pre-, peri-, and postnatal period (13); and the co-inheritance of asymptomatic haplotypes or polymorphisms that combine to generate circuit level excitation/inhibition imbalance (14). The conundrum of an ever-increasing incidence of autism with a lack of clear diagnostic criteria with opaque and wide-ranging etiology underscores the importance of defining circuit dysfunctions lying at the heart of the disorder. To this end, increasing attention has been brought to genes guiding the structural elements of this circuitry, the proteins coded by which are often localized pre- and post-synaptically in the developing brain. Of particular interest regarding the synaptic structural components of brain circuitry are those that rely on AMPA receptor (AMPAr) mediated neurotransmission, which show selective enrichment in the cerebro-cerebellar regions that have increasingly been implicated in ASD pathogenesis (15, 16). This review will focus on two genetic etiologies of autism spectrum disorders, *SHANK3* (encoding the SH3 and multiple ankyrin repeat domains protein) and SYNGAP1 (Synaptic Ras-GTPase activating protein)-related disorders, to highlight how disruption of synaptogenesis in AMPAr-mediated neurotransmission occurs in the pathogenesis of autism in these prototypical neurodevelopmental disorders. Subsequently, a brief look at pharmaceuticals that increase the risk of autism, and how they interact with AMPAr development and function, will subserve the development of a mental model that may allow us to start to bridge the knowledge gap in this enigmatic neurodevelopmental disorder. In particular, what, if any, impact do valproic acid and topiramate, the two drugs that confer the highest risk of autism with *in utero* exposure, have on cerebro-cerebellar circuits and AMPAr neurotransmission will be discussed. Subsequently, how perampanel, a relatively recent addition to the pharmaceutical arsenal and the first non-competitive AMPAr antagonist, interacts with autism spectrum disorder pathophysiology will be evaluated.

Prior to delving into the specific roles of *SHANK3* and *SYNGAP1* in ASD pathogenesis, we will explore AMPAr signaling in the cerebro-cerebellar circuit and discuss briefly how dysfunction in the dendritic compartment at synapses receiving excitatory glutamatergic inputs has been implicated in autism.

## The association of AMPA receptor with cerebro-cerebellar circuit dysfunction in autism

Across mammalian evolution, the expansion of cortical structures couples with the expansion of the cerebellum (17–20). In humans, neocortical expansion is predominantly explained by increase in white matter volume as compared to increases in cerebellar neuron density (20–23). The intimate evolutionary relationship between the cerebellar gray matter and neocortical white matter suggests a crucial role for cerebro-cerebellar network function in neurodevelopment. In fact, cerebro-cerebellar circuit dysfunction has emerged as a key substrate for autism pathogenesis (24, 25) with 1) acquired injury to the cerebellum early in development being identified as a major risk factor for autism diagnosis (1, 26) and 2) decrease in Purkinje cell number (the sole output neuron of the cerebellar cortex) and changes in cerebellar gray and white matter volumes emerging as one of the first neuropathological findings observed in ASD patients (27–29). While the former, acquired injury to the cerebellum, directly implicates cerebellar function in ASD, the cause–effect relationship regarding changes in Purkinje cell number and cerebellar volumes is more difficult to identify, as the tight, interdependent functional relationship between the cortex and cerebellum may implicate primary cortical dysfunction in subsequent cerebellar pathology, as is the case in epilepsy and crossed cerebellar diaschisis (30–33). Finally, emerging research examining *in vivo* network activity using functional magnetic resonance imaging (fMRI) has shown altered connectivity between the cerebellum and various cortical regions in autism (34, 35).

The cell responsible for the massive neuronal density of the cerebellum is the cerebellar granule cell, which accounts for nearly 75% of the total number of neurons in the mammalian nervous system (36). Granule cells receive cortical input from ponto-cerebellar mossy fibers via glutamatergic neurotransmission and project, via parallel fibers, output to Purkinje cells (36, 37). The Purkinje cells then receive excitatory inputs from both granule cell/parallel fiber inputs and olivary projections via climbing fibers (36, 37). This afferent input to the Purkinje cell, which includes pan-cortical input from the ponto-cerebellar projection and peripheral nervous system input from the spino-olivary projection, has been described as “the system with the largest information bandwidth” in the central nervous system (38, 39). Central to this informational throughput is AMPAr-mediated neurotransmission, which is involved in the mediation of each synapse of the afferent projection system: the ponto-cerebellar mossy fiber to granule cell synapse, the olivo-cerebellar climbing fiber to Purkinje cell synapse, and the cerebellar cortical parallel fiber to Purkinje cell synapse (36, 37, 40).

The central position of AMPA receptors in the afferent compartment of the cerebro-cerebellar projection and the importance of cerebellar function to autism pathogenesis combine to highlight the importance of understanding how dysfunction in AMPA receptors in the dendritic compartment is involved in autism. Importantly, cerebellar development occurs primarily in the third trimester of gestation and in early infancy, which is a critical period for autism pathophysiology (1). Crucially, typical granule cell neurotransmission during development has been found to be crucial for normal Purkinje cell, and thus cerebellar, function (41, 42).

**Figure 1:**
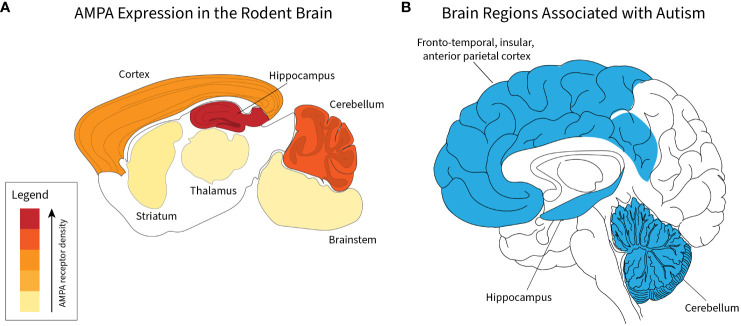
Correlation of brain regions with AMPA receptor expression and autism spectrum disorders. Relative density of AMPA receptors in indicated brain regions based on data presented by Schwenk et al. (44) **(A)**. For indicated regions, relative amount of total AMPA amount is 52% cortex, 23% hippocampus, 17% cerebellum, 3% striatum, 2% thalamus, and 2% brainstem **(A)**. Regions implicated in autism spectrum disorders derived from human studies (45, 46) **(B)**.

In addition to expression in the cerebellum, AMPA receptors are highly expressed in the cortex and hippocampus (43) (**Figure 1A**). Across these brain regions, AMPA receptors associate with a unique profile of interacting proteins and are expressed in different subunit combinations, which likely subserve unique regionally distinct functions (47). Importantly, in addition to the cerebellum, the other two regions with high AMPA receptor expression, the cortex and hippocampus, are known to be functionally involved in autism spectrum disorders (45, 47, 48) (**Figure 1**).

A key question in ASD research is how mutations in what are generally broadly expressed genes or exposure to substances that have broad mechanisms of action on the nervous system can lead to the wide variation of severity and symptomatology seen in autism spectrum disorders. Deficits related to AMPA receptor function, which is broad, fail to elide these concerns at first glance. However, the developmental and functional disruption of the cerebellum, which is increasingly understood to play a crucial role in the very social, emotional, and cognitive domains that are at the heart of deficits that define autism, may subsume this question. The cerebellum plays a key role in the integration of neuro-developmental domains, a role that hinges on the integration of afferent input into the cerebellar cortex and a role that hinges, as we have seen, on intact AMPAr-mediated glutamatergic neurotransmission. Furthermore, ongoing cerebellar function has been found to reflect activity in the prefrontal cortex and hippocampus, further strengthening the possibility that AMPA receptor function is a crucial part of the story in autism pathogenesis (49).

The above discussion alludes to how AMPA receptors may influence circuit level processes in autism pathogenesis, but the majority of work on AMPA receptor dysfunction in autism has focused on how AMPA receptor perturbations are associated with autism on the molecular level (50). To this end, we will discuss how neuronal activity, AMPA receptor signaling, and the dynamic composition of the post-synaptic density (PSD) combine to play a key role in typical neurodevelopment and may be a key site for the pathogenesis of autism. In particular, AMPAr signaling dynamically interacts with the PSD and effects functional synaptic changes—long-term depression (LTD) or long-term potentiation (LTP)—which have been speculated to contribute to disease pathogenesis in ASD.

## AMPA receptors and dendritic compartment physiology

The synthesis of circuit level, cell biological, and molecular processes to account for a syndrome as diverse as autism may be an impossible task. In this review, we will attempt to contextualize AMPA receptors in both the circuit level (as above) and the molecular level through a discussion of two important Mendelian etiologies of autism, disorders related to aberrant *SHANK3* and *SYNGAP1* expression or function. Prior to focusing on these individual proteins, however, a summary defining important features of structural and functional aspects of the synapse as they relate to autism will help contextualize the discussion of the two disorders.

**Figure 2:**
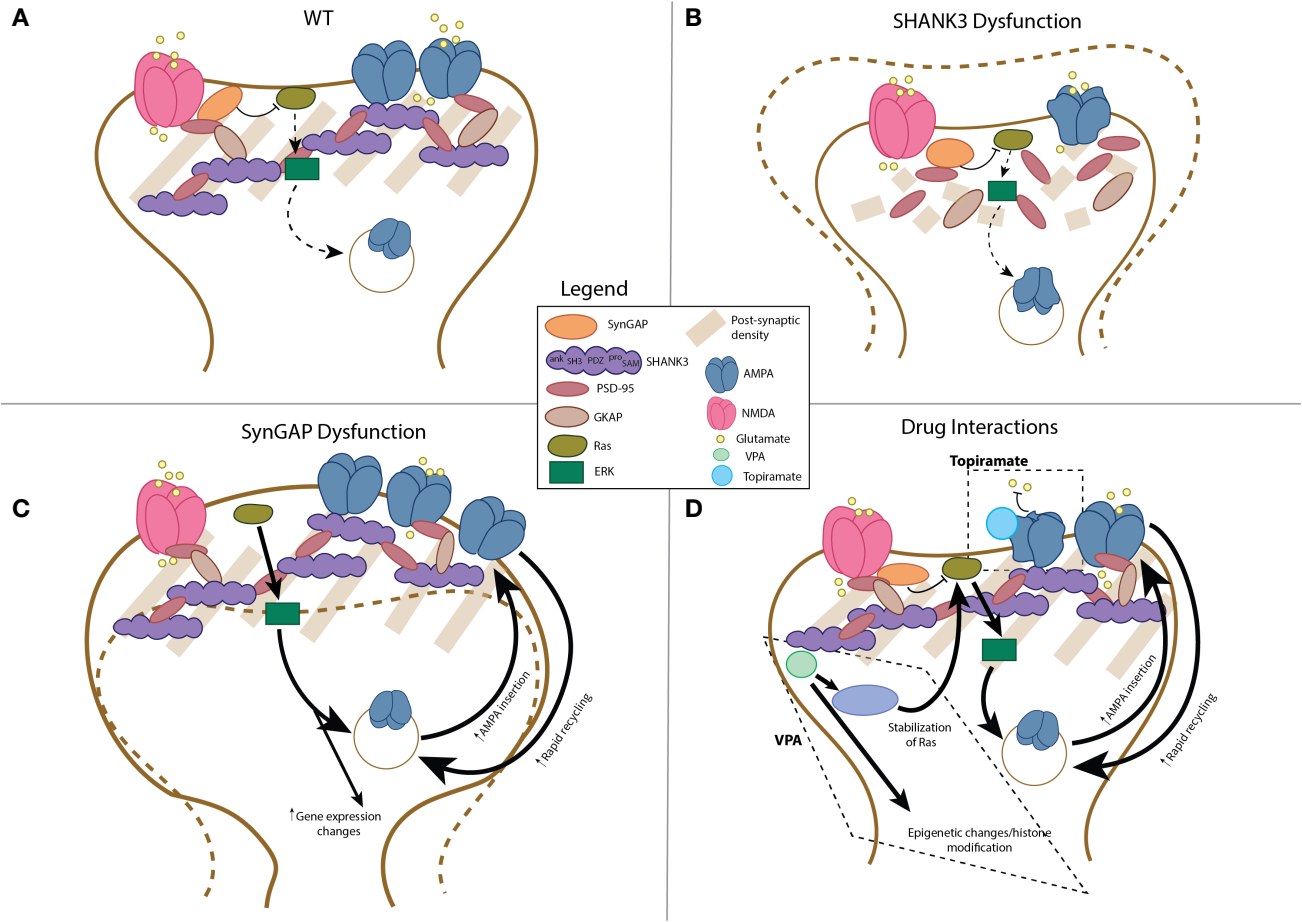
Schematic representation of post-synaptic compartment in AMPAr-associated dendrites in health and disease. Schematic of wild-type dendritic compartment, see also (51) **(A)**. With disruption of SHANK3 protein, structurally diminished post-synaptic compartments, with decreased size of the PSD and depletion of AMPA receptor number is observed (**B**; (52–56)). In contrast, SYNGAP1 dysfunction leads to increased size (“mushrooming”) of the post-synaptic compartment, increased AMPA receptor number, and enhanced Ras-ERK signaling with concomitant changes in gene expression (**C**; (57–60)). The interactions of valproic acid (VPA) and topiramate include the following: for VPA, epigenetic changes related to histone modifications, activation of the Ras-Erk pathway, and changes in expression of AMPAr-related mRNA expression (**D** (61–63)); for topiramate, the primary interaction includes allosteric inhibition of AMPA receptor function (**D**; (64)).

The hallmark of excitatory dendritic spines, the post-synaptic density (PSD), is a highly specialized structure that has evolved to dynamically assimilate neuronal activity through structural modification to strengthen or weaken glutamatergic signaling (51) (**Figure 2A**). The PSD derives its name from its electron dense appearance on electron microscopy (51) and is composed of an abundance of neurotransmitter and transmembrane receptors, scaffold proteins, cytoskeletal elements, and enzymes localized to this structure (51). As will be described, it is thought that SHANK3 and SYNGAP play a key role in PSD structure and function; as a result, their dysfunction, and potentially the effect of various pharmaceuticals on the PSD, may be crucial in the pathogenesis of autism (**Figure 2**).

Dynamic changes at the synapse, which occur as both developmental and adaptive physiological modulations, occur through changes in the efficacy of synaptic transmission. Long-term potentiation (LTP), referring to the ability of brief high frequency stimulation to produce long-lasting strengthening of neurotransmission across individual synapses, is a crucial mechanism by which the nervous system achieves its dynamic adaptability (65). LTP was identified as a key mechanism validating Hebbian plasticity in the middle of the twentieth century (66) and has since become crucial to our understanding of how the nervous system functions as a learning machine despite ongoing discussion regarding experimental considerations and the various forms of the phenomenon (65). In the early 2000s, post-mortem analysis of individuals diagnosed with autism revealed distinct changes in AMPA receptor expression, suggesting alterations in LTP, including broadly increased AMPA receptor mRNA levels and regionally decreased AMPA receptor protein expression in the cerebellum (46). Subsequent studies mechanistically defined one possible causal link by showing that disruption of *UBE3A*, mutations of which are associated with autism and Angelman syndrome, leads to increased internalization of AMPA receptors and decreased LTP (67).

The converse of LTP is long-term depression (LTD), the process by which there is a weakening of synaptic transmission, which can be the result of both pre- and post-synaptic processes (68, 69). LTD has been described as “anti-Hebbian” plasticity and may involve the downregulation of post-synaptic neurotransmitter receptors such as AMPA (68, 69). Interestingly, defects in this process have also been associated with autism. For instance, deletion of *P-Rex1*, a gene responsible for endocytosis of AMPA receptors, results in increased AMPA receptor expression and defects in physiological LTD (70). Mutations of this gene have been associated with autism in patient populations and demonstrated behavioral inflexibility and social deficits in pre-clinical studies (70).

Miniature excitatory post-synaptic currents (mEPSCs) describe the spontaneous discrete release of neurotransmitters at individual synapses and underlie the ongoing function of circuits involving excitatory neurotransmission. Activity-dependent modulation of circuits and pathological circuit dysfunction can thus arise in part from bidirectional alterations in mEPSCs. LTP, characterized by an increase in synaptic strength, leads to larger and more frequent mEPSCs, which reflects the associated increase in synaptic neurotransmission. LTD, on the other hand, is associated with smaller and less frequent mEPSCs reflecting a decrease in synaptic neurotransmission, the inverse of LTP. Accordingly, bidirectional alterations in mEPSCs have been found in pre-clinical models of genes associated with autism in human populations. Pre-clinical studies of a gene found to be associated with autism in patients, *Neurobeachin*, showed significant alterations in dendritic spine morphology and reduction in mEPSCs (71), reflecting how aberrant LTP may be associated with autism. Demonstrating the opposite, pre-clinical studies of a nonsense-mediated decay regulatory gene associated with autism, *Rbm8a*, showed abnormally increased mEPSCs (72), pointing to alterations in LTD being associated with autism.

Together, the physiological modulation of glutamatergic neurotransmission through LTP, LTD, AMPA receptor trafficking, and dynamic regulation of this process at the PSD is critical in normal neural development and functioning. This review seeks to understand how various etiologies of autism—*SHANK3* and *SYNGAP1* related disorders, valproate exposure, and topiramate exposure—may lead to aberrant circuit function through their impact on AMPAr and cerebro-cerebellar circuit function.

## Phelan McDermid syndrome, 22q13 deletion, and other *SHANK3*-related autism etiologies

Autism associated with disruption of *SHANK3* results from a variety of different genetic perturbations, but most commonly from 22q13 deletions (simple deletions and, less commonly, ring chromosome and unbalanced translocations) (73, 74).

Deletions in this region result in a neurological disorder termed Phelan McDermid Syndrome (OMIM 606232) (73, 75),. Individuals with Phelan McDermid Syndrome have characteristic traits including neonatal hypotonia, typically severe developmental delays (particularly in speech/language development), associated ASD, and some minor dysmorphic traits of the face and extremities (75, 76). A number of affected individuals develop other associated conditions including seizures (75, 77), sensory processing differences (78, 79), and significant gastrointestinal disorders (80). Of additional import, individuals with this syndrome often have a broad and unsteady gait, and at times a loss of purposeful hand movements, which suggest cerebellar dysfunction, further implicating cerebro-cerebellar networks (71). There are no characteristic electroencephalographic (EEG) or neuroimaging findings described in affected individuals; however, there is documentation of posterior fossa abnormalities including posterior fossa enlargement or vermian hypoplasia (77). While different associations have produced guidelines for the longitudinal care of children and adults with Phelan McDermid Syndrome (81, 82), no curative therapies (e.g., gene or protein replacement therapies) have thus far been identified.

*SHANK3* is recognized to be strongly associated to ASD in large population studies and is the primary genetic driver of the neurodevelopmental disability seen in Phelan McDermid syndrome (83). Pre-clinical models of *Shank3* disruption have demonstrated similar behavioral and neurological phenotypic traits, including abnormal socialization, repetitive behaviors, and other motoric alterations (52, 84). Other potential associations under investigation include a non-physiological decline in *SHANK3* expression leading to degeneration of excitatory synapses as part of the pathogenesis of Alzheimer’s disease (85).

*SHANK3* encodes the SH3, and multiple ankyrin repeat domains protein 3 (SHANK3), a member of the SHANK family, is encoded in humans by the *SHANK3* gene located on chromosome 22. Highly conserved across species (86), Shank3 is found across human tissues but is most abundantly located in the brain in the PSD as part of the complex network of scaffolding proteins crucial to glutamatergic neurotransmission (86). SHANK3 plays a fundamental role during cerebral development and is highly expressed early during the postnatal course and lifelong for network function; it is a known regulator of the formation and maturation of dendritic spines and integral to synaptic plasticity (53, 73). Although systematic regional abundance of SHANK3 in the human brain has not been evaluated, a recent study has identified high levels of expression of the protein in the cortex, hippocampus, and cerebellum in mouse, with expression predominantly in neuronal layers associated with excitatory neurotransmission (87). Furthermore, white matter evaluations reveal aberrant connectivity in the prefrontal cortex in areas associated with social and cognitive function in both humans and pre-clinical models (88, 89). In relation to effects on cerebellar structure, the first volumetric study in patients with Phelan McDermid found that decreased cerebellar vermal correlated with severity of repetitive behaviors (90). This finding confirmed an earlier studying noting cerebellar vermal hypoplasia in individual cases (91). Whether the cerebellar findings are direct pathological findings related to the dendritic compartment dysfunction that will be described below or are secondary network effects relating to dysfunction in the prefrontal cortex and deep gray cortical remains an open question.

SHANK3 has five interaction domains including a Sterile a motif domain, a proline-rich domain, an src domain, an ankyrin repeats domain, and a PDZ domain, which aid in SHANK3-mediated trafficking of N-methyl-D-aspartate (NMDA) and AMPA receptors (PDZ mediates interactions with NMDA and AMPA receptors via GKAP, but the SH3 domain may directly interact with AMPA receptors and the SAM domain may additionally interact with GKAP and affect NMDA receptors) (53–55) (**Figure 2B**). As mentioned above, disruption of AMPAr function has broad influences on processes implicated in autism pathogenesis including synapse development, LTP, and LTD. SHANK3 has also been found to influence dendritic spine morphology through a mechanism of NMDA receptor expression and AMPA receptor [rapid] recycling (92) (**Figure 2B**). Furthermore, mutations in *Shank3* have been shown to result in depletion of AMPA receptors and a change in morphology of the PSD (52, 56). Different mouse and rat models have also demonstrated altered dendritic spine morphology resulting in decreased dendritic spine density and increased complexity of arborization, dendritic length, and surface area (55, 56). Correlating these microstructural changes to the brain volumetric and network level structural and functional seen in Phelan McDermid and other ASDs will be of great interest moving forward.

In pre-clinical models, the distinct morphological and electrophysiological changes in synaptogenesis and synaptic plasticity, including the role of AMPAr conduction/receptor function, particularly at a post-synaptic level have been studied in greater detail.

One group used a *Shank3* knockout (KO) adult mouse model to show altered AMPAr-mediated mEPSCs at the PSDs of neurons in the anterior cingulate cortex and striatum with decreased frequencies (suggestive of fewer functional synapses), irrespective of changes to NMDA receptor or presynaptic function (93, 94). Structurally, this was associated with a decreased dendritic spine density and altered spine width (93). Electrophysiological evaluation again demonstrated not only lower AMPA receptor-mediated mEPSC frequency but also amplitude, and decreased AMPA/NMDA receptor ratio, suggestive of weakened excitatory inputs to this important cortical structure in inducing LTP (93). These findings have been replicated in similar studies targeting specific exon deficits in both heterozygous and KO mice in hippocampal neurons (95, 96). In one study, human *SHANK3* heterozygous neurons engrafted onto mouse prefrontal cortex demonstrated similar findings, suggesting similar electrophysiological differences (reduced mEPSC amplitude and decreased AMPA/NMDA receptor ratio) and structural changes (impaired dendritic arborization), sustained several months post-engraftment (97). In another study, a *Shank3* heterozygous mouse model with suggested structural changes in the AMPA receptor subunit GluR1 in the hippocampus demonstrated a decrease in AMPA-receptor-mediated field excitatory potentials as demonstrated by a reduction in the input/output (I/O) slope, and decreased mEPSC amplitude. However, mEPSC frequency was observed to be increased, suggesting a compensatory presynaptic alteration (55). Overall, these studies suggest a predominant impact on LTP with relatively unimpaired long-term depression (LTD).

Other studies have suggested functionally similar results, however in association with different interactions and influences. In one homozygous mouse model with a specific alteration to exon 21 of *Shank3*, decreased NMDA/AMPA receptor ratios are suggested to occur as a result of decreased NMDA receptor responses (altered evoked/spontaneous synaptic transmission with unaffected mEPSC amplitude) (97). In another mouse study, alteration on metabotropic glutamate receptor mGluR5 and HOMER receptosome is hypothesized to result in downstream alteration of both AMPA and NMDA receptor function effectively “freezing” synapse sensitivity to the physiological cues regulating LTP and LTD (98).

Antibody-mediated SHANK3 protein function has also demonstrated the impact of SHANK3 disruption on AMPA receptor function during synaptogenesis, both in a direct and indirect manner (through NMDA receptor alteration). Several mouse and rat models employing anti-SHANK3 antibodies at a hippocampal neuronal level have demonstrated structural dysregulation of NMDA and AMPA receptor clustering (99) and disrupted exocytosis of AMPA receptors via Rho-GAP interacting CIP4 homolog (Rich2) associations induced by, and later influencing of, LTP (100). Induced depolarization via potassium chloride of embryonic rat cortical neurons demonstrated that prolonged depolarization leads to increased glutamate effect via NMDA receptors in turn reducing SHANK3-GluA1 interactions and AMPA receptor function (101).

In model organisms, *in vivo* restoration of protein function using various platforms has shown recovery of AMPAr-mediated dysfunction, perhaps paving the way for future therapies in *SHANK3*-related autism. In one instance, selective restoration of SHANK3 in the anterior cingulate cortex of adult mice led to partial restoration of mEPSC frequency and full restoration of mEPSC peak amplitudes and I/O responses and NMDA/AMPA receptor current ratios (93). In hippocampal neurons, disrupted mGlu-HOMER to SHANK3 interaction was hypothesized to lead to impaired NMDA receptor activation (in turn decreasing NMDA/AMPA receptor post-synaptic current ratio) bypassed through GluN2B-HOMER restoration, improved structure (scaffolding), and rescued NMDA/AMPA receptor current ratios (98).

In other adult KO mouse models, striatal restoration of function using conditional knock-ins demonstrated recovery in mEPSC currents (102). Indirectly, one *Shank3*-deficient mouse model underwent chemogenic activation in pyramidal neurons of the prefrontal cortex via designer receptor exclusively activated by designer drugs (DREADDs) (103), to rescue NMDA receptor function. The immediate restoration demonstrated no influence in AMPA receptors, but after 1–2 h of activation of DREADDs, AMPA receptor EPSCs significantly increased in wild-type and deficient mice (104).

Separately, intraperitoneal use of IGF-1 in hemizygous adult mice reverse specific AMPA receptor deficits (as observed in mean slope of I/O function) resulting in restored hippocampal LTP after 2 weeks (105).

Together, the combined clinical and basic science investigations into SHANK3-associated autism spectrum disorders reveal alterations in cerebro-cerebellar network function, with underlying disruption on the level of synapse structure and function. The predominant impact of *SHANK3* dysfunction as it relates to AMPAr includes a decrease in dendritic synapse size and aberrant LTP. As we will see in the next section, *SYNGAP1*-associated autism spectrum disorders result in seemingly opposite molecular and cellular phenotypes, yet produce similar network and clinical phenotypes.

## SYNGAP1-developmental and epileptic encephalopathy

SYNGAP1-related intellectual disability, or SYNGAP1-related developmental and epileptic encephalopathy (SYNGAP1-DEE, OMIM 612621), typically presents with early life developmental delays without frank regression, in association with exam findings including hypotonia or strabismus (106). In addition—and similar to Phelan McDermid—one observed symptom (and possible biomarker) is ataxic/wide-based gait, again possibly suggestive of cerebellar dysfunction and implicating cerebro-cerebellar networks, which affects over 50% of cases (107). Some of these phenotypic traits had, previously, led to inclusion of SYNGAP1-DEE patients in the category of “Angelman-like” syndrome (108). There are, however, no pathognomonic/syndromic traits, and paraclinical evaluations such as neuroimaging are non-contributory (109). Approximately one-half of all patients develop autism spectrum disorders, but a number of other patients develop additional behavioral difficulties, such as impulsivity or aggression. More than four-fifths of individuals develop epilepsy, with characteristic traits of myoclonic–atonic events, and eyelid myoclonia with atypical absence seizures, often associated with mastication (110–113). EEG traits described include generalized epileptiform discharges and episodic occipital runs of delta slowing (109). Despite growing interest in its research as an incrementally important gene in neurodevelopment, and the ever-improving care in epilepsy due to *SYNGAP1* haploinsufficiency, there are no curative treatments to date.

Since the initial publication on SYNGAP1-related conditions from initial descriptions of unrelated individuals with non-syndromic intellectual disability (114), advances have been made in the characterization of associated genetic changes with loss of function by heterozygous truncating mutations in the majority of cases (89%) and deletions to the 6p21.3 locus in a minority (11%) (106).

*SYNGAP1* encodes a synaptic ras-GTPase-activating protein, which is a neuron-specific RasGAP protein expressed predominantly in the PSD of the mammalian forebrain as a mediator of excitatory glutamatergic neurotransmission by way of suppression of RAS-ERK pathways (negative regulation of RAS GTPase). Its interactions impact the organization of molecular complexes within the dendritic spines, thus mediating synapse formation, maturation, and plasticity (57, 58) (**Figure 2C**). Dysregulation, or disruption, of *SYNGAP1* results in neurons with premature dendritic maturation with abnormal dendritic spines (“mushrooming”) (59) (**Figure 2C**). The regulation and disruption of plasticity occurs in regions of the brain that mediate processes of memory and cognition, particularly impactful during early brain development (59, 60). To date, no studies have reported structural or functional network abnormalities using brain imaging in SYNGAP1-DEE patients; however, it should be noted that epilepsy does reflect gross abnormalities in brain networks.

Beyond its function as a post-synaptic scaffolding protein, the understanding of the role of SYNGAP1 has emanated from its regulation of glutamatergic NMDA and AMPA receptors. SYNGAP1 is involved in the “gating” of the NMDA receptor’s ability to influence AMPA receptor trafficking; the stimulation of NMDA receptors in turn induces Rho-kinase phosphorylation of SYNGAP1 and inactivation of the Ras-ERK pathway, ultimately resulting in decreased AMPA receptor trafficking and post-synaptic membrane insertion (115–117). Overexpression of SYNGAP1 in mouse and rat models is known to diminish AMPA receptor insertion, surface expression, and associated mEPSCs (118). This regulatory process is crucial in the process of regulating LTP during synaptic plasticity and synaptic bouton formation (119). As a whole, however, the effect of *SYNGAP1* variations and isoforms is incompletely understood; at least one mouse model has studied the effect of alternate splicing or alternative promoter usage upon *Syngap1* in forebrain neurons, resulting in variable AMPA receptor mEPSC amplitude and frequency, and even inducing “opposing” effects (120).

*SYNGAP1* haploinsufficiency and SYNGAP protein disruption have extensively been studied in human and non-human neuronal models of disease. The effect of altered synaptogenesis and excitatory/inhibitory homeostasis generally has been demonstrated to lead to cross-species developmental, behavioral, and neurologic alterations (among others), including epilepsy, aberrant behaviors, and cognitive impairment (59, 121). Additional research has highlighted the potential role of *SYNGAP1* mutations in sensory processing changes and enteric motility differences (122, 123).

The role that SYNGAP1 plays in synaptogenesis, synaptic plasticity, and involvement of AMPA-receptor-mediated circuitry have been increasingly studied in rodent models. Heterozygous mice model studies have observed a change in the Ras-ERK interaction and resulting recruitment of AMPA receptors in the post-synaptic membrane along thalamocortical pathways, medial prefrontal cortex, and the hippocampus, during early brain development. This results in increased local AMPA/NMDA receptor currents, affecting the “unsilencing” of synapses to induce LTP. As a result, AMPA and NMDA receptor ratios (as a function of mEPSC measures) increase and stabilize, indicative of early maturation and a restricted period of plasticity (impaired LTP due to precocious “unsilencing”). In one study, a subsequent compensatory increase in mGluR-mediated potentiation of LTD, while no changes in NMDA receptor deactivation kinetics in another, was observed (60, 124). The excitation/inhibition (E/I) imbalance during synaptic plasticity results in a “form of stabilized cortical hyperexcitability”, corroborated in studies of *ex vivo* heterozygous mouse models of pyramidal mPFC neurons. In this, mEPSC amplitude and frequency were observed to be increased, whereas miniature inhibitory post-synaptic current (mIPSC) amplitude was observed to be decreased, suggesting that heterozygous *Syngap1*+/− mice have an elevated E/I ratio and an increased I/O slope/curve (125).

Accelerated dendritic maturation at a hippocampal level has been observed as early as postnatal day 14: an increased AMPA/NMDA receptor ratio (suggestive of premature acquisition of adult level AMPA receptors) and subsequent increased post-synaptic AMPA receptor mEPSC amplitude and frequency (without reported change in resting membrane potentials), with (as prior noted) possible compensatory increase in mIPSCs, were documented by one group (126).

Similar findings have been observed in homozygous KO mice models. Both structural changes—early maturation of spines with wide heads (“mushrooming”), large PSD clusters, including NMDA NR1 receptor and GluR1 (AMPAr) clusters—and electrophysiological changes—higher mEPSC frequency (suggestive of more glutamate release) and higher amplitude (larger physiological response clusters)—have been documented (127). Transfecting these neurons with SYNGAP1-GFP results in the return of mEPSC frequencies back to wild-type levels, whereas mEPSC amplitudes decrease below WT levels (118).

At least one human-induced pluripotent stem cell KO model has demonstrated similar findings. CRISPR-generated neurons demonstrate increased GluA1 structures and larger post-synaptic structures. When observing developmental timepoints, immature cells have earlier higher synaptic activity, alongside larger mEPSC events (amplitude and frequency are increased), whereas at later developmental timepoints, there is less of a corresponding increase in mEPSCs when compared to WT cells. Overall, this suggests early maturation and precocious onset of “coordinate network bursting behavior” (128).

Direct disruption of protein expression (by way of small interfering RNA, siRNA) and protein function (via SYNGAP1-blocking peptides affecting its phosphorylation and effective relation between NMDA and AMPA receptors), absent pathogenic loss of function to *Syngap1* gene, in rodent hippocampal and forebrain neurons have also demonstrated effects on mEPSC frequencies (increase) and variable effect on mEPSC amplitude (unchanged or increased) (118, 129).

An interesting exploration of SYNGAP1 function beyond the direct effect on cortical excitatory synapses is in its effect on inhibitory interneurons. One recent study has explored the disruption of medial ganglionic eminence interneurons, subsequently affecting firing properties of hippocampal CA1 neurons. A mice model with a conditional allele affecting a loxP site demonstrated effect on fast spiking inhibitory interneurons (FS-INs), affecting the I/O relationship and slope of I/O curve of AMPA receptor mEPSCs upon electrical stimulation, alongside a small increase in mEPSC amplitude and potency, without reported effect on NMDA receptors. This appears to affect short-term dynamics of plasticity without effect on regular spiking interneurons. There is also decreased spontaneous mIPSCs, suggesting a defect in synaptic inhibition. This overall increase in AMPA-receptor-mediated synaptic inputs suggests network hyperexcitability (altered inhibitory function) propending a risk for cognitive impairment and seizures, mediated by AMPA receptors (130).

At least one non-human model has sought to rescue protein function and limit downstream effects of *Syngap1* insufficiency. The role of perampanel, a non-competitive antagonist of AMPA receptors, has been explored to modulate specific hippocampal interneurons responsible for modulation of gamma oscillations observed on EEG, in particular, regarding its role in affecting GluA2 AMPA receptor subunit dynamics that appear affected during sleep in *Syngap1* heterozygous mice (130).

Together, SHANK3 and SYNGAP1 research has revealed how bidirectional dysregulation of AMPA receptor regulation and function can converge on autistic phenotypes in these two important etiologies of the disorder (**Figure 2**). With aberrant SHANK3 protein function, there is structural loss of PSD integrity, decrease in dendrite size and number, and aberrant LTP. Conversely, with *SYNGAP1* disruption, mushrooming of the synapse is seen with basal cortical hyperexcitability. In parallel to these physiological and microstructural changes, network level studies reveal decreased brain volumes in patients with disrupted *SHANK3* expression, while SYNGAP1-DEE patients have generally normal imaging with high penetrance of epilepsy. A drawback to functional brain imaging studies, which might be useful to further parse these network level effects, is that they are difficult to conduct in patients with intellectual disability. Having reviewed the mechanistic underpinnings of AMPAr dysfunction in two genetic autism etiologies, the review will now turn to two acquired etiologies of autism that result from *in utero* drug exposure.

## Autism associated with prenatal anti-seizure medication exposure

While it is increasingly understood that injury to key brain regions early in life may contribute to subsequent diagnosis of ASD (1), here, we will focus on drug exposures during pregnancy that predispose to ASD putatively through alterations in neurotransmission in the developing brain. Consonant with the theory that excitation/inhibition imbalance may be an important factor in the development of ASD (14), exposure to anti-seizure medications during gestation has been shown to predispose to ASD (131). Anti-seizure medications often have complex “dirty” mechanisms of action, affecting various aspects of neurotransmission simultaneously (132). These include effects on action potentials propagation through ionotropic channel modulation, effects on inhibitory neurotransmission through GABA modulation, effects on excitatory glutamatergic neurotransmission, and more chronic effects on transcriptional and translational programs (132). A recent population based study using the Nordic database was the largest to look at associations of anti-seizure medication exposure during gestation with subsequent development of ASD, examining the outcomes of nearly 4.5 million children (131). This study found a robust and dose-dependent association between two monotherapies during pregnancy and subsequent diagnosis of autism in the child, topiramate and valproic acid (131).

Valproic acid (VPA) administration during pregnancy has long been associated with significantly increased risk for childhood diagnosis of autism (133–135). Despite this long-standing association, however, a clear mechanistic understanding of the role of VPA exposure in autism pathogenesis remains to be found. Part of the difficulty in the mechanistic understanding of VPA-associated autism arises from the population-based nature of the association studies, thus capturing a cohort of patients with autism arising from *in utero* exposure to VPA has been difficult. Towards an understanding of the network effects of VPA exposure, the impact of VPA use on brain volume was evaluated based on exposure to the drug (136). In this study, VPA was found to be associated with diminished gray matter in the cerebellum, frontal cortex, and hippocampus, which correlates to the cerebro-cerebellar network connectivity that has previously been discussed and with areas of increased AMPA receptor expression (**Figure 1**). This association was confirmed in a rat model, where *in utero* VPA exposure revealed decreased cerebellar volume, decreased Purkinje cell number, and abnormal functional connectivity of cerebro-cerebellar networks (137).

Mechanistic evidence of the effect of VPA on neurodevelopment has relied on pre-clinical studies, which have supported the association of VPA exposure with autism, forming the basis for a key murine model of ASD (61). Hypotheses of the mechanism of action related to ASD pathogenesis include the ability of VPA to alter the gene expression profile of neurons through histone modifications or its effect on patterning through modification of *Wnt* signaling (61). Provocatively, VPA also acts on the Ras-ERK pathway through indirect beta-catenin activation, which then acts directly on ERK signaling (62). Furthermore, several recent studies have looked more directly at the effect of prenatal VPA exposure on glutamatergic neurotransmission. One study found that VPA leads to altered GluA2 in the hippocampus and prefrontal cortex secondary to deficits in RNA editing of this AMPAr subunit (62) (**Figure 2D**). A second study looking at gene expression changes, also in the hippocampus and prefrontal cortex, found that VPA exposure *in utero* led to differential expression of mRNA and miRNA of many post-synaptic genes, including some involved in LTD and AMPA receptor complex (63). More recently, it was found that VPA exposure leads to increased AMPA receptor expression, while the resulting social deficits could be rescued with AMPA receptor antagonists (138). The effect of VPA on the developing nervous system is broad, and many direct and indirect effects on neurotransmission and neurodevelopment likely play into its role in increasing ASD risk. More work is needed to understand the effects of VPA on the AMPAr circuit and developmental synaptogenesis, especially given the recent findings of interactions with AMPAr subunit expression and Ras-ERK signaling, which suggest mechanistic similarity to SYNGAP1-DEE.

While the association between VPA exposure and autism has been known for several decades and have led to strong guidelines about use in women of child-bearing age, the risks of topiramate exposure to the developing brain remain undersold. Despite the strong association between topiramate exposure during pregnancy and subsequent ASD diagnosis (131, 139–142) adequate guidance for women about exposure risk is lacking in clinical practice (143). Furthermore, the pathogenic mechanism of topiramate in ASD has not been well studied through clinical or pre-clinical examination. At the time of writing, a PubMed search for “topiramate” and “autism” or “neurodevelopment” yields 50 results with a single pre-clinical publication while replacing “topiramate” with “valproate” yields over 900 publications of which over 500 are preclinical.

Several studies have evaluated the effect of topiramate on AMPA receptor function (64, 144, 145). One early study showed that topiramate depresses activity across glutamate receptors, although this study failed to differentiate between a specific effect on AMPA vs. kainate receptors (the latter being another glutamatergic receptor subtype) (145). A subsequent study showed that topiramate has an allosteric effect on AMPA receptor inhibition on neurons cultured from brain regions implicated in autism (64) (**Figure 2D**). The likely allosteric nature of this inhibition was confirmed by a more recent study that did not reveal direct modulation of AMPA receptors by topiramate using *in vitro* methods (144). More research is needed to understand the impact of topiramate on neurodevelopment, which is a pressing issue, as topiramate remains a common medication used in women of child-bearing age. Furthermore, the development of a pre-clinical model of topiramate-exposure-associated ASD may shed further light on the contribution of synaptic pathogenesis in autism.

Given that AMPA receptors are a key mediator of glutamatergic neurotransmission involved in many functions, the AMPAr antagonist, perampanel (originally approved as an anti-seizure medication), has been used in a wide variety non-epilepsy or epilepsy associated conditions, including in autism with associated epilepsy (146). Several studies have looked at the efficacy of perampanel in rare epilepsies, including epilepsy associated with SYNGAP1-DEE (147–149). One study focused on rare epilepsy, which often feature significant developmental phenotypes, and revealed high efficacy for perampanel (148). Two more-focused studies (one a small case series the other a case report) evaluated the impact of perampanel on behavior in autism with associated epilepsy (147, 149). The case series revealed that 7 out of 17 patients showed improvement on autism behavioral assessments, including two out of five patients that did not show reductions in seizures (149). The case report evaluated the response of cortical gamma frequencies to low dose perampanel treatment in a patient with SYNGAP1-DEE associated autism and epilepsy, a perspective based on a pre-clinical investigation (147, 150). The group found that there was improvement in the EEG signatures of cortical dysfunction and improvement in behavior but failure in seizure improvement. Furthermore, as a case report, they were unable to evaluate whether the behavioral improvements were a result of perampanel or concomitant behavioral interventions (147). Nonetheless, the findings in these studies point to the exciting possibility that AMPAr antagonism may have some beneficial effects on behavior in some autism etiologies. However, it is important to note that in other cases where AMPAr hypofunction may be a key pathogenic mechanism, such as in SHANK3-associated autism, AMPAr antagonism may worsen behavioral phenotypes. This contrast highlights the importance of mechanistically understanding autism etiologies on a case by case basis. Furthermore, understanding how different etiologies converge on a network level, for example, on cerebro-cerebellar networks, may allow for treatment of autism with cryptic etiologies leveraging non-invasive neuromodulation and that adaptive capacity of brain networks. In fact, a recent headway has been made showing that transcranial direct current stimulation, targeting both frontal and cerebellar nodes of the network, may show promise in the treatment of autism-associated behavioral problems (151, 152).

## Concluding thoughts

Autism is a prevalent neurodevelopmental disorder whose pathophysiology remains poorly understood (1–5). Diverse pathogenetic mechanisms have been investigated including extensive studies on molecular, structural, and network effects. There remain broad inconsistencies in findings, often defined by the limited understanding of potential etiologies and pathogenetic mechanisms of developmental brain dysfunction (153). Recent identification of certain groups of neurogenetic disorders with high penetrance of autism and autistic symptoms in affected individuals has shed light on specific subsets of pathophysiological mechanisms and their intersection. In this review, we have attempted to approach ASD from the perspective of dysfunction in AMPAr-mediated neural circuits, with a focus on cerebro-cerebellar connectivity and the synaptic dysfunction emerging in two prototypical synaptopathies, Shank3/Phelan McDermid Syndrome and SYNGAP1-DEE. In these disorders, alteration in the function of the post-synaptic compartment, including with the PSD, AMPA receptor subunits, and the subsequent effect on synaptic physiology (LTP and LTD), have been independently associated with the clinical presentation of ASD. Elaborating on the concept of exposure and non-genetically mediated (or co-mediated) dysfunction, review of two commonly used anti-seizure medications with the greatest propensity to predispose children with a history of gestational exposure to autism seeks to understand how these neuromodulatory drugs might influence AMPAr function and contribute to development of autism. Understanding AMPAr-mediated neural circuit dysfunction may be clinically relevant in the care of child-bearing patients especially as ongoing efforts continue towards the development of targeted therapeutics for epilepsy and beyond. These efforts are especially important in light of the exponentially increasing rates of ASD (6–8). Finally, we briefly addressed how AMPAr antagonism may alleviate autistic symptoms, especially in cases of autism with co-morbid epilepsy, perhaps highlighting the importance of understanding precise etiologies of autism.

The review is limited by the complexity and promiscuity of nervous system development and physiology, with given molecules and mechanisms being adapted by different cell types and pathways throughout development. As a result, the focus on AMPA receptors has come at the expense of a discussion of AMPA’s partner in excitatory neurotransmission, NMDA, and neglect of the flip side of excitation/inhibition, GABA-ergic neurotransmission. Furthermore, in the acquired etiologies discussed, VPA and topiramate exposure, the absence of studies on patients who have autism as a result of these exposures makes the implication of AMPAr in these etiologies highly speculative.

Accordingly, part of the difficulty of understanding autism arises from the complexity of the disorder itself from an etiological, pathophysiological, and diagnostic perspective, all compounded by the inherently interconnected nature of the nervous system. The neuromodulatory roles of SHANK3, SYNGAP1, valproic acid, and topiramate during critical developmental periods are multifaceted, and thus, pinning their effect on a single aspect of neural circuit function is impossible. Even more difficult is how opposite effects on a single circuit may converge on the unifying phenotype of autism. As detailed, SHANK3 and SYNGAP1 have opposing effects on the structure and function of the post-synaptic compartment: syndromes associated with disruption of *SHANK3* lead to decreases in synapse size, integrity, and AMPAr function, while SYNGAP1-DEE shows increased synapse size, AMPAr number, and excitability. Similarly, while valproic acid stimulates Ras-ERK-mediated pathways (mimicking the Ras-ERK effects of loss of SYNGAP1 function), topiramate acts as an allosteric inhibitor of AMPA receptors. Unifying these observations is the timing of the implied dysfunction, concomitant with early brain development (*in utero* in the case of drug exposures and throughout brain development in the case of the genetic etiologies) and the convergence on cerebro-cerebellar networks. Thus, contextualizing AMPA receptor function—and neurotransmission more broadly—not only in the sub-second time frame that is normally used but also in developmental time may be a crucial component to understanding the how circuit abnormalities intersect with autism spectrum disorders.

## Author contributions

AJ-G: Conceptualization, Writing – original draft, Writing – review & editing. MN: Visualization, Writing – review & editing. JG: Conceptualization, Writing – original draft, Writing – review & editing.
